# The Role of the Multifunctional BAG3 Protein in Cellular Protein Quality Control and in Disease

**DOI:** 10.3389/fnmol.2017.00177

**Published:** 2017-06-21

**Authors:** Elisabeth Stürner, Christian Behl

**Affiliations:** Institute of Pathobiochemistry, University Medical Center of the Johannes Gutenberg University MainzMainz, Germany

**Keywords:** proteostasis, protein quality control, selective macroautophagy, BAG3, neurodegenerative disorders

## Abstract

In neurons, but also in all other cells the complex proteostasis network is monitored and tightly regulated by the cellular protein quality control (PQC) system. Beyond folding of newly synthesized polypeptides and their refolding upon misfolding the PQC also manages the disposal of aberrant proteins either by the ubiquitin-proteasome machinery or by the autophagic-lysosomal system. Aggregated proteins are primarily degraded by a process termed selective macroautophagy (or aggrephagy). One such recently discovered selective macroautophagy pathway is mediated by the multifunctional HSP70 co-chaperone BAG3 (*BCL-2-associated athanogene 3*). Under acute stress and during cellular aging, BAG3 in concert with the molecular chaperones HSP70 and HSPB8 as well as the ubiquitin receptor p62/SQSTM1 specifically targets aggregation-prone proteins to autophagic degradation. Thereby, *BAG3-mediated selective macroautophagy* represents a pivotal adaptive safeguarding and emergency system of the PQC which is activated under pathophysiological conditions to ensure cellular proteostasis. Interestingly, BAG3-mediated selective macroautophagy is also involved in the clearance of aggregated proteins associated with age-related neurodegenerative disorders, like Alzheimer’s disease (tau-protein), Huntington’s disease (mutated huntingtin/polyQ proteins), and amyotrophic lateral sclerosis (mutated SOD1). In addition, based on its initial description BAG3 is an anti-apoptotic protein that plays a decisive role in other widespread diseases, including cancer and myopathies. Therefore, in the search for novel therapeutic intervention avenues in neurodegeneration, myopathies and cancer BAG3 is a promising candidate.

## Introduction

Cell viability relies on an intact and functional proteome. The basic requirement for the cellular function of a protein is the proper folding in its native conformation. Under physiological conditions, misfolding of a protein is often caused by genetic mutations, its incorrect transcription/translation or its inefficient biogenesis. However, environmental or intracellular stress and also changes due to aging highly increase the amount of misfolded proteins. Such proteins often expose hydrophobic regions which facilitate the formation of potentially toxic protein aggregates. Therefore, irreversibly damaged or misfolded, dysfunctional proteins have to be efficiently eliminated. The stability and metabolism of the proteome is tightly controlled by the cellular protein quality control (PQC). In cellular PQC, chaperone systems cooperate as a network and function together with protein transport and degradation systems to ensure the integrity of the proteome and protein homeostasis (proteostasis). In recent years, comprehensive studies identified that in addition to the ubiquitin-proteasome system selective autophagy as a lysosomal degradation pathway fulfills essential functions in PQC. An imbalance or failure of proteostasis is linked to aged-related neurodegenerative disorders, such as Alzheimer’s disease (AD), Parkinson’s disease (PD), Huntington’s disease (HD), or amyotrophic lateral sclerosis (ALS).

This review will focus on the role of the BAG3-mediated selective macroautophagy pathway as well as of the anti-apoptotic co-chaperone BAG3 (*BCL-2-associated anthanogene 3)* itself in cellular PQC and in severe diseases, including cancer and myopathies. In particular, it will highlight the activity of BAG3-mediated selective macroautophagy in the context of neurodegenerative disorders.

## Proteostasis Network

**FIGURE 1:**
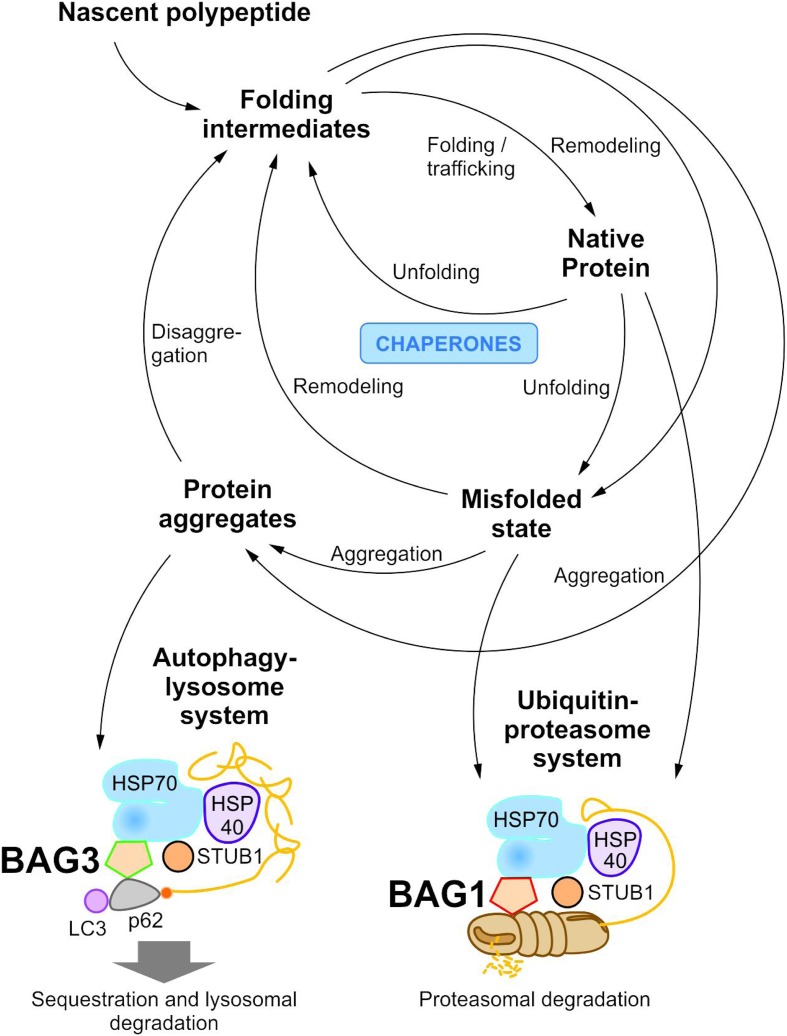
The cellular proteostasis network and its degradation systems. In cellular protein quality control, molecular chaperone systems function in concert with protein transport and degradation systems to ensure cellular proteostasis. In addition to the ubiquitin-proteasome system, the autophagy-lysosome system mediates the disposal of degradation-prone substrates.

To maintain protein homeostasis, the cellular protein quality control provides an elaborated, tightly regulated network consisting of several different chaperone systems and two different protein degradation systems (**Figure 1**) (Hartl and Hayer-Hartl, 2002; Bukau et al., 2006; Hartl et al., 2011; Saibil, 2013; Hipp et al., 2014; Labbadia and Morimoto, 2015). Due to their ability to identify and bind to unstructured and hydrophobic regions of non-native proteins with high affinity, molecular chaperones have a key function in cellular PQC and proteostasis (Hartl and Hayer-Hartl, 2002; Hartl et al., 2011; Kim Y.E. et al., 2013; Saibil, 2013). On the one hand, they monitor and assist the proper folding of unfolded nascent proteins into their native and functional conformation; on the other hand, when proteins have not yet reached or cannot reach their native state at all, they promote either their refolding or direct them to degradation. The degradation of non-native proteins is thereby mediated by the ubiquitin-proteasome system (see The Ubiquitin-Proteasome System) or the autophagy-lysosome system (see The Autophagy-Lysosome System). Under stress conditions, such as proteotoxic stress or heat stress, the expression of various molecular chaperones is enhanced (constitutive vs. induced expression) and, therefore, they were also referred to as heat shock proteins (HSPs). The group of HSPs contains several structurally unrelated protein superfamilies, such as the HSPA (HSP70) system or the HSPB (small HSP) system in humans (Kampinga et al., 2009). All HSP systems also exhibit constitutively expressed members like HSC70 in the HSP70 system. In cellular PQC, protein homeostasis is basically ensured by the interplay of these different HSP chaperone systems. Thereby, substrates bind to them both directly and sequentially. For instance, the HSP70 chaperone system (see The HSP70 Chaperone System) is implicated in various PQC functions (e.g., substrate identification in macroautophagy) and thereby mostly cooperates with members of other HSP chaperone systems, like the HSPB chaperones.

The HSPB chaperones, also referred to as small HSPs in mammals, are low-molecular mass chaperones which are ubiquitously expressed (Vos et al., 2008; Kampinga et al., 2009). The structure of HSPBs is characterized by a highly conserved α-crystalline domain flanked by a more variable N-terminal sequence and often a short, variable C-terminal tail (Vos et al., 2008). The α-crystalline domain mediates many inter- and intra-molecular interactions, including the formation of HSPB dimers, the functional unit of HSPB activity. HSPB dimers in turn can interact with each other, thereby forming oligomers. Besides their chaperone function, HSPBs are able to stabilize the cytoskeleton and exhibit anti-apoptotic activity (Boncoraglio et al., 2012). HSPB chaperones prevent irreversible protein aggregation in an ATP-independent manner. For further processing (e.g., refolding or degradation) of bound substrates, they require members of ATP-dependent HSP chaperone systems, such as the HSP70 chaperone system. Thus, HSPBs form multi-chaperone complexes, like the HSPB8-HSP70 complex (Carra et al., 2008b). The activity and even substrate specificity of HSPBs seem to be regulated by their phosphorylation and oligomerization state.

### The HSP70 Chaperone System

The human HSP70 (HSPA) chaperone family comprises 13 members which are expressed constitutively (e.g., HSPA8 = HSC70) or stress-induced (e.g., HSPA1A = HSP70/HSP72) (Kampinga et al., 2009). The highly conserved HSP70 chaperones are ubiquitously expressed and mainly function in protein quality control by monitoring the folding of newly synthesized proteins and by directing misfolded proteins to refolding, disaggregation, or degradation (Mayer and Bukau, 2005; Young, 2010; Kim Y.E. et al., 2013; Saibil, 2013). HSP70 chaperones consist N-terminally of an ATPase domain, also called nucleotide-binding domain (NBD), and C-terminally of a substrate-binding domain (SBD); the two domains are connected via a highly conserved hydrophobic linker region. HSP70 substrate binding and release are controlled by ATP in an ATP-consuming cycle (Mayer and Bukau, 2005; Young, 2010; Hartl et al., 2011; Kim Y.E. et al., 2013; Saibil, 2013). The nucleotide state of the ATPase domain thereby affects the confirmation of the SBD and in this manner regulates the affinity of the SBD for non-native proteins. In the ATP-bound configuration, substrates can enter the SBD of the HSP70 chaperone. The following hydrolysis of ATP to ADP stabilizes the interaction between the SBD and the substrate, resulting in a stable substrate binding by HSP70. The subsequent nucleotide exchange as well as the rebinding of a new ATP molecule triggers the release of the substrate.

To carry out its function in cellular PQC, the HSP70 chaperone collaborates with other chaperones (e.g., HSPB chaperones) and also acts in concert with a number of co-chaperones and co-factors (e.g., the E3-ligase STUB1, HIP, HOP) (Mayer and Bukau, 2005; Young, 2010; Kim Y.E. et al., 2013; Saibil, 2013). Both co-chaperones and co-factors regulate the activity of HSP70 by modulating its ATP-consuming cycle. Important co-chaperones of HSP70 are the HSP40 proteins and the nucleotide exchange factors (NEFs). The HSP40 proteins (J-domain proteins or DNAJs) represent a diverse, large chaperone family with more than 40 specialized members which can target HSP70 to specific sites and functions (Kampinga and Craig, 2010; Young, 2010; Dekker et al., 2015). HSP40s contain a conserved J-domain that interacts with the N-terminal ATPase-domain of HSP70. They not only recognize HSP70 substrates and assist them to enter the HSP70 chaperone, but also stimulate the ATPase activity of HSP70. As their name already indicates, NEFs stimulate the nucleotide exchange and the slow intrinsic ADP release after ATP hydrolysis within the HSP70 chaperone (Mayer and Bukau, 2005; Young, 2010; Bracher and Verghese, 2015). Thereby, they promote the release of the HSP70 substrate and facilitate the restart of the ATPase-cycle. The structurally unrelated family of eukaryotic NEFs includes the HSP110 proteins and the BAG (*BCL-2-associated athanogene*) proteins. HSP110s are structurally related to HSP70 and represent the most abundant NEFs in the eukaryotic cell (Schuermann et al., 2008). Presumably, they act in cooperation with HSP70 and HSP40s as holdases in protein disaggregation. For a detailed overview of characteristics and functions of BAG proteins the authors also refer to a recent review (Behl, 2016). Briefly, the human BAG protein family contains six members, namely BAG1, BAG2, BAG3 (BIS/CAIR), BAG4 (SODD), BAG5 and BAG6 (Scythe/BAT3) (Takayama et al., 1999; Takayama and Reed, 2001). All BAG proteins feature a conserved BAG domain in their C-terminal region; BAG5 possesses three additional BAG domains. It was shown that except for BAG5 all members of the BAG family physically interact with HSP70 via their BAG domain, suggesting a function of BAG proteins as HSP70 targeting factors. In addition to their BAG domain, all BAG proteins have further protein–protein interaction domains, like a PxxP region, a WW domain or a ubiquitin-like (UBL) domain, which allow binding to diverse cellular proteins. BAG proteins are implicated in a variety of cellular key pathways. Interestingly, BAG1 and BAG3 were recently identified as new key players in the cellular PQC (see BAG3-Mediated Selective Macroautophagy) (Gamerdinger et al., 2009; Minoia et al., 2014).

### The Ubiquitin-Proteasome System

In eukaryotic cells the ubiquitin-proteasome system (UPS) is a highly conserved degradation system that mediates the ubiquitination and the subsequent proteasomal degradation of intracellular proteins (Hershko and Ciechanover, 1998; Pickart, 2001; Kerscher et al., 2006; Finley et al., 2012).

Within ubiquitination, the 8.5 kDa, highly conserved protein ubiquitin is covalently attached to a lysine residue of the degradation-prone protein via an isopeptide bond. Ubiquitination is an ATP-dependent, sequential enzymatic process which is catalyzed by the enzymes E1, E2, and E3 of the ubiquitin conjugation cascade (Hershko and Ciechanover, 1998; Pickart, 2001; Kerscher et al., 2006; Ye and Rape, 2009). Upon its ATP-consuming activation by the ubiquitin-activating enzyme E1, ubiquitin is transferred to a ubiquitin-conjugating enzyme E2 by forming a high energy thioester bond. In concert with an E3-ligase (E3-enzyme/ubiquitin ligase) the E2 enzyme subsequently mediates the covalent conjugation of ubiquitin to the target. The ubiquitin conjugation machinery is hierarchically organized with two E1s, approximately 50 E2s and approximately 1000 E3s in mammals. The various E3s are responsible for the specific recognition of the numerous UPS substrates. Ubiquitin itself contains seven lysine residues (K6, K11, K27, K29, K33, K48, K63); to each of these lysine residues an additional ubiquitin molecule can be conjugated, thereby forming poly-ubiquitin chains (Komander and Rape, 2012; Akutsu et al., 2016). Depending on the exact lysine residue, poly-ubiquitin chains of different linkage type and structural topology are formed and act as signals for various processes (Peng et al., 2003; Meierhofer et al., 2008; Komander, 2009; Xu et al., 2009; Komander and Rape, 2012). K48-, K63-, and K11-linked poly-ubiquitin chains are the most abundant and best studied poly-ubiquitin chains in the cell (Peng et al., 2003; Meierhofer et al., 2008; Komander, 2009; Xu et al., 2009). K48- as well as K11-linked poly-ubiquitin chains were demonstrated to assign the substrate for proteasomal degradation (Chau et al., 1989; Chen and Pickart, 1990; Hershko and Ciechanover, 1998; Thrower et al., 2000; Jin et al., 2008; Xu et al., 2009). However, except for K63-linked poly-ubiquitin chains, all other linkage types of poly-ubiquitin chains may also mediate the proteasomal degradation of the respective substrate (Peng et al., 2003; Meierhofer et al., 2008; Komander, 2009; Xu et al., 2009; Ye and Rape, 2009). In contrast, K63-linked poly-ubiquitin chains are mainly involved in non-proteolytic processes, such as DNA repair or signal transduction (Hofmann and Pickart, 1999; Deng et al., 2000; Chen and Sun, 2009; Erpapazoglou et al., 2014).

Within the UPS the degradation of poly-ubiquitinated proteins is performed by the 26S proteasome, a barrel-shaped highly conserved multi-protein complex (Voges et al., 1999; Saeki and Tanaka, 2012; Livneh et al., 2016). The 26S proteasome consists of the 20S core particle which is flanked by the 19S regulatory particles. The proteolysis of poly-ubiquitinated proteins is mediated by the 20S core particle that acts as a threonine protease with trypsin-, chymotrypsin- and caspase-like activities. The 19S particles function in the recognition, the unfolding and the processing of the substrates (Finley, 2009). Notably, only unfolded substrates can be processed by the 26S proteasome; non-dissociable aggregates cannot enter the narrow channel of the 26S proteasome. Recently, there is strong evidence that these substrates are degraded by the autophagy-lysosome system.

### The Autophagy-Lysosome System

Autophagy is an evolutionarily highly conserved process in eukaryotes that degrades cytosolic constituents by means of the lysosome (Johansen and Lamark, 2011; Parzych and Klionsky, 2014; Klionsky et al., 2016). In every cell, the autophagic machinery is active at a low basal level to remove long-lived proteins, protein aggregates or damaged organelles; however, under certain stress conditions, for instance nutrient starvation, growth factor withdrawal or hypoxia, the activity of autophagy can be induced. The induction as well as the process of autophagy are sophisticatedly regulated by autophagy-related genes/proteins (Atgs/ATGs) which are conserved from yeast to human (Klionsky et al., 2003; Mizushima et al., 2011; Jin and Klionsky, 2014; Klionsky and Schulman, 2014; Wesselborg and Stork, 2015). By forming functional protein complexes, ATG proteins are able to control autophagy at different steps. In addition, different non-ATG proteins were shown to be implicated in the regulation and process of autophagy, including mTOR (*mammalian/mechanistic target of rapamycin*), AMPK (*AMP-activated protein kinase*), AKT/PKB (*protein kinase B*), AMBRA1 (*activating molecule in BECN1-regulated autophagy protein 1*), BCL-2 (*B-cell lymphoma/leukemia-2*), DFCP1 (*double FYVE domain-containing protein 1*), or VPS34 (*vacuolar protein sorting protein 34*) (Wesselborg and Stork, 2015). In mammals, so far the following six autophagy-regulating protein clusters are known: (1) the ULK1 (*Unc-51-like kinase 1*)-ATG13-FIP200 (*FAK family kinase-interacting protein of 200 kDa*)-ATG101 protein kinase complex, (2) the PtdIns3K (*phosphatidylinositol 3-kinase*) class III complex containing the core proteins VPS34, VPS15 (*vacuolar protein sorting protein 15*) and Beclin 1, (3) the PtdIns3P (*phosphatidylinositol 3-phosphate)*-binding WIPI (*WD repeat domain phosphoinositide-interacting protein*)/ATG18-ATG2 complex, (4) the multi-spanning transmembrane protein ATG9A, (5) the ubiquitin-like ATG5/ATG12 system and (6) the ubiquitin-like ATG8/LC3 conjugation system (Mizushima et al., 2011; Jin and Klionsky, 2014; Wesselborg and Stork, 2015).

To date, three primary types of autophagy are generally distinguished: mircroautophagy, chaperone-mediated autophagy (CMA) and macroautophagy (Boya et al., 2013; Parzych and Klionsky, 2014; Klionsky et al., 2016). In microautophagy, degradation-prone components are directly eliminated by the invagination of the lysosomal membrane (Mijaljica et al., 2011). In CMA, proteins carrying the pentapeptide lysosome-targeting motif KFERQ are selectively recognized by the HSP70 chaperone and targeted to the lysosomal membrane; subsequently, after their binding to the integral lysosomal membrane protein LAMP-2A and their unfolding substrates of the CMA are translocated into the lysosome (Massey et al., 2004). In contrast, substrates of the macroautophagy pathway are sequestered away from the lysosome by forming double-membrane vesicles, the so-called autophagosomes.

**FIGURE 2:**
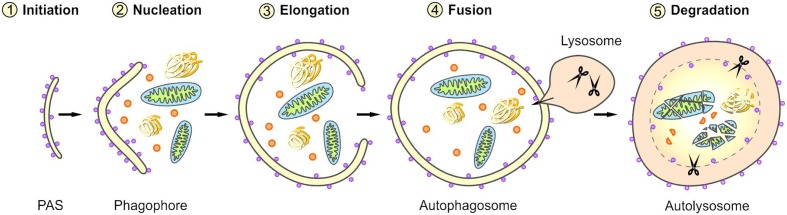
Scheme of selective macroautophagy. Macroautophagy represents a complex multi-step process. After the initiation of macroautophagy (1: Initiation) a phagophore is formed at the phagophore assembly site (PAS) (2: Nucleation). Subsequently, the phagophore expands by recruiting membranes derived from different intracellular sources (3: Elongation) and thereby sequesters the cargo. The closing of the phagophore results in the formation of a mature autophagosome. Following its transport to the lysosome, the autophagosome fuses with the lysosome (4: Fusion), generating an autolysosome. Within the autolysosome, the enclosed material is degraded (5: Degradation).

Macroautophagy is a complex, tightly controlled multi-step process (depicted schematically in **Figure 2**) (Feng et al., 2014; Parzych and Klionsky, 2014; Klionsky et al., 2016). Following the initiation of macroautophagy, a cup-shaped phagophore is formed at the phagophore assembly site (PAS), a process also called nucleation. This phagophore subsequently expands by recruiting membranes from different intracellular sources. The elongation of the phagophore results in the engulfment of the degradation-prone substrates and thereby in the formation of a mature double-membraned autophagosome. The autophagosome is then transported to the lysosome and its outer membrane fuses with the lysosomal membrane, generating an autolysosome. The macroautophagic pathway often merges with the endocytic pathway; therefore, in some cases the autophagosome may primarily fuse with an early or late endosome (amphisome) before its fusion with the lysosome. Within the autolysosome, the inner membrane of the autophagosome and the enclosed cellular material are degraded by lysosomal proteases and the resulting components are exported back into the cytoplasm via lysosomal permeases. The origin of membranes for the biogenesis of autophagosomes are still controversially discussed and under intensive investigation (Lamb et al., 2013; Carlsson and Simonsen, 2015; Kern et al., 2015). Different sources probably contribute to *de novo* synthesis and completion of the autophagosomal membrane and thus the formation of autophagosomes has been associated with diverse cellular compartments, such as the endoplasmic reticulum (ER), the Golgi apparatus, mitochondria, recycling endosomes, or the plasma membrane.

For quite some time, autophagy was regarded as a non-selective bulk and robust degradation process (Kopitz et al., 1990). Based on a more detailed characterization of the macroautophagy process and especially on the identification of further players in macroautophagy, today it is known that macroautophagy represents a very specific and highly selective process (Rogov et al., 2014; Stolz et al., 2014; Khaminets et al., 2016). The selectivity of macroautophagy is thereby ensured and monitored by special factors, the so-called autophagy receptor (Rogov et al., 2014; Khaminets et al., 2016). Autophagy receptors, such as p62/SQSTM1 (*sequestosome 1*) or NBR (*neighbor of Brc1*), are able to direct degradation-prone substrates to the autophagic system by their simultaneous interaction with the cargo and autophagy proteins in the autophagosomal membrane, such as the MAP1LC3 protein (*microtubule-associated protein light chain 3;* LC3) (Pankiv et al., 2007; Slobodkin and Elazar, 2013). In the last years, many different selective macroautophagy pathways were identified and named after their cargo, for instance aggrephagy (aberrant or disease-related protein aggregates), xenophagy (intracellular pathogens) or mitophagy (mitochondria) (Rogov et al., 2014). Degradation-prone material is targeted to selective macroautophagy in an ubiquitin-dependent, but also in an ubiquitin-independent manner. Therefore, ubiquitin-dependent and ubiquitin-independent pathways exist in selective macroautophagy (Stolz et al., 2014; Khaminets et al., 2016). In addition, the various selective macroautophagy pathways are controlled by auxiliary factors and adaptors which partly are cell type-, age-, or disease-specifically expressed. For instance, the HSP70 co-chaperone BAG3 or the deacetylase HDAC6 were shown to represent such auxiliary factors (Kawaguchi et al., 2003; Gamerdinger et al., 2009; Ouyang et al., 2012).

The dysfunction or the deregulation of autophagy is associated with a variety of human disorders, including lung, liver and heart disease, neurodegenerative diseases, myopathies, cancer, or metabolic diseases (Choi et al., 2013; Nixon, 2013; Jiang and Mizushima, 2014; Schneider and Cuervo, 2014).

## The Multifunctional HSP70 Co-chaperone BAG3

BAG3 (*BCL-2-associated athanogene 3*), also referred to as BIS (*BCL-2 interacting death suppressor*) or CAIR-1 (*CAI stressed-1*), was identified in 1999 by a yeast two-hybrid screen for proteins interacting with the HSC/HSP70 ATPase domain (Takayama et al., 1999). It belongs to the family of BAG co-chaperones which interact specifically with the ATPase domain of the HSP70 chaperone via their conserved C-terminal BAG domain (Takayama and Reed, 2001; Kabbage and Dickman, 2008). BAG3 is a multifunctional protein which is implicated in the regulation of a variety of cellular processes, such as apoptosis, development, cytoskeleton arrangement and selective macroautophagy. Thereby, it plays a decisive role in the development of widespread diseases, including cancer, myopathies, and age-related neurodegenerative disorders (Behl, 2011, 2016; Gamerdinger et al., 2011a; Rosati et al., 2011; Knezevic et al., 2015).

### BAG3 Expression and Its Modulation

BAG3 is evolutionarily highly conserved in mammals (Wada et al., 2006); BAG3 orthologs in mouse, rat and human show a significant high homology not only at protein level, but also at gene level. The BAG3 protein comprises a molecular weight of 75 kDa (Lee et al., 1999). In addition to this full-length gene product of the *bag3* gene, a C-terminal truncated form of BAG3 with a molecular weight of about 40 kDa has been detected in neuronal synaptosomes (Bruno et al., 2008, 2014). However, this shorter BAG3 variant is not yet characterized in detail.

The 75 kDa BAG3 is predominantly localized in the cytoplasm under physiological conditions. A nuclear localization of a small BAG3 subpool could be observed in some cell types, such as glial cells or pancreatic carcinoma cells (Gentilella and Khalili, 2009; Rosati et al., 2011). Under acute stress or upon viral infection, BAG3 alters its subcellular distribution; for instance, the co-chaperone translocalizes to the nucleus or is found to be enriched juxtanuclearly/perinuclearly in aggregates/inclusion bodies (Kyratsous and Silverstein, 2008; Gamerdinger et al., 2009, 2011b; Jin et al., 2015). Considering its expression pattern in human tissues, BAG3 is ubiquitously expressed, however, to different degrees. Especially, a high constitutive BAG3 expression is reported in muscle cells (heart and skeletal) and in cells of different cancer types (e.g., lymphoid or myeloid leukemias, neuroblastomas, prostate carcinomas, ovarian and breast carcinomas, glioblastomas or pancreatic carcinomas) (Liao et al., 2001; Homma et al., 2006; Rosati et al., 2007a; Gentilella and Khalili, 2011; Felzen et al., 2015; Sherman and Gabai, 2015). In non-transformed cells, like epithelial or retinal cells, the expression of BAG3 can be induced by stress, for example caused by oxidants (e.g., H_2_O_2_ or hydroxyl nonenal), high temperature, heavy metals, HIV infection, or proteasome inhibition (e.g., by MG132 or bortezomib) (Pagliuca et al., 2003; Bonelli et al., 2004; Rosati et al., 2007b; Wang et al., 2008; Gamerdinger et al., 2009). An increased cellular BAG3 level was additionally verified during cellular aging in neuronal cells as well as in lung fibroblasts (Gamerdinger et al., 2009). Furthermore, BAG3 levels are elevated after treatment with small compounds like the disaccharide trehalose, the thiol molecule pyrrolidine dithiocarbamate (PDTC) or the natural derivative of cinnamaldehyde, 2′-hydroxycinnamaldehyde (HCA) (Song et al., 2010; Lei et al., 2015; Nguyen and Kim, 2017).

The *bag3* gene expression is under the control of diverse endogenous physiological factors which bind to specific sequences within the promoter region of *bag3*. The enhanced BAG3 level detected upon stress or application of compounds results mostly from the induction of BAG3 expression by the heat shock transcription factor 1 (HSF1) (Franceschelli et al., 2008; Song et al., 2010; Nguyen and Kim, 2017). Thereby, HSF1 interacts with two heat shock-responsive elements (HSEs) within the *bag3* promoter. Moreover, the expression of the *bag3* gene was shown to be activated in a NF-κB-dependent indirect manner after proteotoxic or heat stress (Nivon et al., 2012; Rapino et al., 2015). Apart from these prominent transcription factors, it is known that the oncogene WT1 (*Wilm’s tumor 1 protein*) transcriptionally induces the expression of BAG3 and that the transcription factor AIbZIP (*androgen-regulated protein androgen-induced bZIP*) also upregulates BAG3 expression (Ben Aicha et al., 2007; Cesaro et al., 2010). In response to the fibroblast growth factor 2 (FGF2), BAG3 expression is positively modulated in neuroblastoma and neuronal progenitor cells; in this context, it could be demonstrated that the activation of *bag3* in neuroblastoma cells is mediated by stimulation of the transcription factor EGR-1 (*early growth response protein 1*) (Gentilella et al., 2008; Gentilella and Khalili, 2010). Noteworthy, in glial cells BAG3 is even able to auto-regulate its transcription by binding to its own promoter (Gentilella and Khalili, 2009).

**FIGURE 3:**
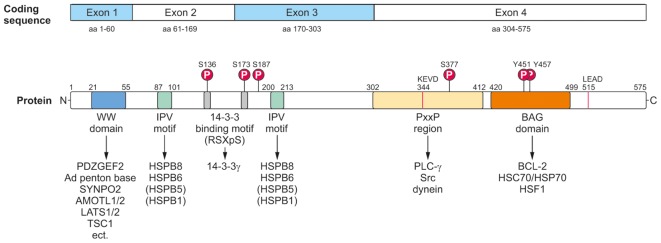
Modular protein structure of BAG3. BAG3 is encoded by four exons (Coding sequence). The BAG3 protein comprises various characteristic amino acid motifs/regions and domains which permit the interaction of BAG3 with numerous proteins involved in many cellular key pathways. In addition to its conserved BAG domain, BAG3 contains a conserved WW domain, two IPV motifs, two 14-3-3 binding motifs and a PxxP region. Moreover, a caspase cleavage site was identified in the PxxP region (^344^KEVD) of BAG3 and at its very C-terminus (^515^LEAD). The BAG3 protein is suggested to be phosphorylated at the indicated serine and tyrosine residues.

At the protein level, BAG3 can be modulated by post-translational modifications and hereby not only its interaction with other proteins, but also its activity and function might be changed. To date, the best, but still not sufficiently studied post-translational modification of BAG3 is its phosphorylation (**Figure 3**). Upon EGF (*pro-epidermal growth factor*) stimulation in human breast cancer cells Doong et al. (2000) demonstrated a tyrosine phosphorylation of the conserved BAG domain (tyrosine 451 or 457) of BAG3. Moreover, it could be shown that BAG3 can be phosphorylated at serine 187 by the protein kinase C delta (PKCδ); this modified form of BAG3 then triggers the epithelial-mesenchymal transition and invasiveness of thyroid cancer cells (Li et al., 2013). The binding of the 14-3-3γ protein, a recently newly identified player in aggresomal targeting, to the co-chaperone BAG3 depends on the BAG3 phosphorylation status; by mutation of serine at position 136 or 173 in the phosphoserine-containing 14-3-3 binding motif (RSXpS) of BAG3 the interaction between 14-3-3γ and BAG3 is diminished or even abolished (Xu et al., 2013). Iorio et al. (2015) discovered an increased tyrosine phosphorylation of BAG3 mediated by the focal adhesion kinase (FAK) upon glucose stimulation in pancreatic beta cells. This phosphorylation of BAG3 causes a loss of the interaction between BAG3 and SNAP-25 which in turn allows the formation of a t-SNARE complex and the release of insulin. Other putative phosphorylation sites of BAG3 have been uncovered via phosphoproteomic studies (Dephoure et al., 2008; Ge et al., 2010; Xue et al., 2014); for instance, it was reported that after proteasome inhibition by bortezomib the serine residue 377 of BAG3 is increasingly phosphorylated in multiple myeloma cells (Ge et al., 2010). To further elucidate the relevance of phosphorylation, but also of other post-translational modifications (e.g., ubiquitination) for BAG3 and its pathways, a systematic analysis of the post-translational modifications of BAG3 is absolutely essential. Interestingly, BAG3 was found to be ubiquitinated by the ubiquitin ligase STUB1 and eventually degraded by the proteasome upon autophagosome formation in myocytes (Arndt et al., 2010). Furthermore, Virador et al. (2009) detected a basal ubiquitination of BAG3 that is enhanced after caspase cleavage under staurosporine stress. The poly-ubiquitinated BAG3 was shown to be then degraded by the proteasome (Virador et al., 2009). These findings suggest a regulation of BAG3 by proteasomal proteolysis.

The human *bag3* gene is located on the long arm of chromosome 10 at position 26.11 (10q26.11) (uniprot). Mutations in the *bag3* gene are associated with the development of severe diseases, such as myofibrillar myopathy or dilated cardiomyopathy (see Impact of BAG3 on Myopathies) (Selcen et al., 2009; Arimura et al., 2011; Norton et al., 2011; Villard et al., 2011; Lee et al., 2012). The knockout of the *bag3* gene in mice (by retroviral insertion) leads to a fulminant postnatal myopathy followed by death before 4 weeks of age (Homma et al., 2006). *bag3*-deficient mice are characterized by a postnatal retarded growth, a disproportionate small body, a pulmonary edema and an abnormal sarcomere and skeletal muscle morphology. Moreover, a degeneration of myocardial and skeletal muscle fibers and an increased cardiomyocyte apoptosis were observed. The myogenic differentiation of *bag3*-deficient mice seems to proceed normally and thus it is assumed that the early lethality of these mice originates from respiratory and heart failure (Homma et al., 2006). Youn et al. (2008) developed also mice with a homozygous disruption of the *bag3* gene, however by applying a *cre-loxP* system and thereby precisely targeting the exon 4 of *bag3* (encoding the BAG domain and the PxxP region). However, these mice display a phenotype which is slightly different from the phenotype described by Homma et al. (2006). They die 3 weeks after birth and their detailed analysis revealed small and irregular muscle fibers, a spleen and thymus hypoplasia, hypoglycemia, a general nutritional impairment and the complete inhibition of the BAG3 protein synthesis (Youn et al., 2008). In contrast to Homma et al. (2006), Youn et al. (2008) found no evidence for a massive apoptosis in the myocardium of their *bag3*-deficient mice.

### Protein Interaction Domains/Motifs and Interaction Network of BAG3

The BAG3 protein has a multi-modular domain structure that permits the interaction of BAG3 with a variety of proteins involved in all kinds of cellular pathways (**Figure 3**) (McCollum et al., 2009). As all members of the BAG protein family, the BAG3 protein exhibits the highly conserved BAG domain in its C-terminal region (Takayama et al., 1999; Doong et al., 2002). Via this ∼80 aa domain, BAG3 is able to bind not only to the anti-apoptotic protein BCL-2 and the ATPase domain of the HSC/HSP70 chaperone, but also – as recently shown- to the heat shock factor HSF1 (Lee et al., 1999; Doong et al., 2002, 2003; Jin et al., 2015). In addition to its BAG domain, the human 575 aa BAG3 protein contains three other characteristic amino acid motifs/regions and domains: a WW (tryptophan-tryptophan) domain, two IPV (isoleucine–proline–valine) motifs and a PxxP (proline-rich) region. Its N-terminally located WW domain interacts with proline-rich repeats of proteins, such as the guanine nucleotide exchange factor 2 (PDZGEF2), the adenovirus (Ad) penton base protein, synaptopodin-2 (SYNPO2), the YAP/TAZ inhibitors LATS1/2 or AMOTL1/2 or the tuberous sclerosis 1 (TSC1) protein (Gout et al., 2010; Iwasaki et al., 2010; Ulbricht et al., 2013b; Kathage et al., 2017). Two conserved IPV motifs were identified N-terminally and in the middle of BAG3 and mediate the binding of BAG3 to the small heat shock proteins HSPB8 (HSP22) as well as HSPB6 (HSP20) and to a smaller extend also to HSPB5 (αB-crystallin) and HSPB1 (HSP27) (Carra et al., 2008a; Fuchs et al., 2009; Rauch et al., 2017). The PxxP motif/repeat of BAG3 represents a binding site for proteins containing a SH3 (Src homology 3) domain, like phospholipase C gamma (PLC-γ) or Src, and for the motor protein dynein (Doong et al., 2000; Gamerdinger et al., 2011b; Colvin et al., 2014). Between its two conserved IPV motifs, BAG3 possesses two phosphoserine-containing 14-3-3 binding motifs (RSQS^136^ and RSQS^173^) which are crucial for its interaction with the 14-3-3γ protein (Xu et al., 2013). Furthermore, a conserved caspase cleavage site is located in the PxxP region (^344^KEVD) of BAG3 and at its very C-terminus (^515^LEAD) (Virador et al., 2009). Between these multiple structured protein–protein interaction domains and motifs, the BAG3 protein exhibits several regions of predicted structural disorder. Such disordered regions within proteins are far from being functionless; for instance, they were shown not only to be “linker” segments between structured protein domains, but also to be subjected to post-translational modifications and to mediate protein–protein interactions (Babu, 2016).

In HeLa cells an analysis of the BAG3 interactome was performed by using the quantitative immunoprecipitation combined with knockdown (QUICK) method and proteome microarrays (Chen et al., 2013). The identified putative BAG3 interaction partners are proteins with diverse functions, among them proteases, transferases, transcription factors, and signaling molecules. The high number of already identified and still unknown different BAG3 interactors suggests that the co-chaperone BAG3 has a pivotal regulatory impact on many determining biological processes, including apoptosis, cell proliferation, development, cytoskeleton arrangement, cell adhesion, cell motility, viral replication, and selective macroautophagy. Due to its role in so many cellular key pathways, it is not surprising that a dysfunction or a deregulation of BAG3 has a devastating effect on cells and tissues.

### Impact of BAG3 on Myopathies

Naturally occurring mutations in the human *bag3* gene are mostly associated with the development of myopathies (**Table 1**) (Selcen, 2010; Knezevic et al., 2015). In 2009, Selcen et al. (2009) discovered that the single missense mutation of proline to leucine at codon 209 (Pro209Leu) in exon 3 of *bag3* (626C > T) causes a severe type of myofibrillar myopathy (MFM). Patients harboring this single allelic substitution displayed a fulminant muscular dystrophy with rapidly progressive limb and axial muscle weakness in early childhood, in the second decade of life accompanied by respiratory insufficiency and the development of cardiomyopathy, often resulting in early death. This phenotype of human BAG3-related MFM was verified by other studies; additionally, an axonal neuropathy with giant axons was observed in some cases (Odgerel et al., 2010; Jaffer et al., 2012; Lee et al., 2012; Konersman et al., 2015; Kostera-Pruszczyk et al., 2015). Notably, the heterozygous Pro209Leu BAG3 mutation, which is situated in the second of the two conserved IPV motifs of BAG3, seems to be a spontaneous mutation that occurs *de novo* in the early embryonic development (Odgerel et al., 2010). Moreover, other mutations of the *bag3* gene, for instance Arg218Trp, Leu462Pro, a 10-nucleotide mutation in exon 4 or a deletion of exon 4 of *bag3*, were reported to be causative of (familial) dilated cardiomyopathy (DCM) (Arimura et al., 2011; Norton et al., 2011; Villard et al., 2011; Chami et al., 2014; Feldman et al., 2014; Franaszczyk et al., 2014; Toro et al., 2016). DCM is characterized by severe heart failure, often combined with a sudden cardiac death. Interestingly, increased levels of extracellular BAG3 (secreted by stressed cardiomyocytes) and of BAG3 antibodies were identified in sera of patients with heart failure (De Marco et al., 2013, 2014). Due to the possibility of detection in the serum of patients, BAG3 is discussed as a suitable prognostic factor for heart failure (Gandhi et al., 2015).

**Table 1:** Mutations in BAG3 implicated in myopathies.

Human pathology	Mutation within BAG3	Reference
**Myofibrillar myopathy (MFM)**	Pro209Leu in exon 3	Selcen et al., 2009; Odgerel et al., 2010; Jaffer et al., 2012; Lee et al., 2012; Konersman et al., 2015; Kostera-Pruszczyk et al., 2015
	Pro209Gln in exon 3	Semmler et al., 2014
**Dilated cardiomyopathy (DCM)**	Deletion of exon 4	Norton et al., 2011
	Arg71Trp in exon 2	
	His109Arg in exon 2	
	Ala262Thr in exon 3	
	Arg477His in exon 4	
	Arg90X in exon 2	
	Arg123X in exon 2	
	Arg218GlyfsX89 in exon 3	
	Glu455Lys in exon 4	Villard et al., 2011
	Val468Met in exon 4	
	Arg309X in exon 4	
	Gln251ArgfsX56 in exon 3	
	Ser385GlnfsX56 in exon 4	
	Arg395GlyfsX48 in exon 4	
	Arg218Trp in exon 3 Leu462Pro in exon 4	Arimura et al., 2011
	10-nucleotide deletion in exon 4 (with fs and X after 13aa)	Feldman et al., 2014
	Deletion of exons 3-4	Franaszczyk et al., 2014
	Glu455Lys in exon 4	
	Tyr451X in exon 4	
	Gln353ArgfsX10 in exon 4	
	Gly379AlafsX45 in exon 4	
	Ser249X Arg309X	Chami et al., 2014
	His243ThrfsX64	Toro et al., 2016

*Various missense and nonsense mutations as well as several frameshift mutations with premature stop codons in the coding exons of BAG3 were identified to be causative of myofibrillar myopathy or dilated cardiomyopathy. Fs, frameshift; X, premature stop codon*.

As already mentioned, BAG3 is strongly expressed in skeletal and heart muscle cells and co-localizes with Z-disks. Patients featuring BAG3 mutations are characterized by disrupted Z-disks, the degeneration and disorganization of myofibrils (Selcen et al., 2009; Arimura et al., 2011). At the molecular level, the Z-disk-associated protein BAG3 maintains the Z-disk integrity and muscle contractility by different mechanisms: firstly, BAG3 together with HSP70 ensures the structural stability of filamentous actin (F-actin) by promoting the interaction between HSP70 and the actin capping protein beta 1 (CapZβ1) as well as by facilitating the cellular localization of CapZβ1 (Hishiya et al., 2010). Secondly, it was shown that BAG3 also regulates the level of the actin-crosslinking protein filamin in Z-disks (Arndt et al., 2010). Upon mechanical stress or muscle exercise a multi-chaperone complex consisting of BAG3, HSP70, HSPB8 and the ubiquitin ligase STUB1 targets damaged filamin for degradation to lysosomes by an autophagic process, termed chaperone-assisted selective autophagy (CASA). In addition, BAG3 seems to support autophagosome formation by interacting with synaptopodin-2 and to initiate autophagy by interfering with mTORC1 regulation (by binding to TSC1, a component of the mTORC1 inhibiting complex) (Ulbricht et al., 2013a,b, 2015; Kathage et al., 2017). Concomitantly with CASA, BAG3 also induces the transcription of filamin by engaging the YAP/TAZ inhibitors LATS1/2 and AMOTL1/2 (Ulbricht et al., 2013b). By balancing degradation and synthesis of filamin, BAG3 guarantees the maintenance of the Z-disks in myocytes (Ulbricht et al., 2013a). Moreover, BAG3 was found to co-localize with Ca^2+^ channels and to bind to β1-adrenergic receptors in ventricular myocytes, thereby affecting contractility and calcium homeostasis (Feldman et al., 2016).

### Role of BAG3 in Cancer

The HSP70 co-chaperone BAG3 was found to be upregulated in many human cancers of various origins, for instance in melanomas, glioblastomas, or pancreatic adenocarcinomas. In many cases, BAG3 promotes the survival, the growth and the invasiveness of primary tumors and/or provides resistance to chemotherapy. This anti-apoptotic activity of BAG3 depends on its ability to bind to numerous cellular proteins which are involved in major biological processes. A vast number of studies addressing the anti-apoptotic, pro-survival activity of BAG3 in cancer were published. In the following, only some recent studies are exemplarily listed to illustrate the significance of BAG3 for cancer biology.

By analyzing biopsies of pancreatic ductal adenocarcinoma (PDAC) patients, enhanced intracellular BAG3 expression was found to correlate with poorer survival (Rosati et al., 2012). Interestingly, BAG3 could be also detected in sera of PDAC patients (Falco et al., 2013). Recently, it has been reported that pancreatic ductal adenocarcinoma cells are able to release BAG3 which activates macrophages via its binding to the receptor IFITM-2 (*interferon-induced transmembrane protein 2*) (Rosati et al., 2015). The BAG3-mediated activation of macrophages through the PI3K and the p38 MAPK signaling pathways results in secretion of further cell proliferation-stimulating factors, proposing a function of extracellular BAG3 in tumor development. Indeed, the application of an anti-BAG3 monoclonal antibody impaired tumor growth and metastatic spreading in mouse models (Rosati et al., 2015). High BAG3 levels were claimed to contribute to the maintenance of glioblastoma stem cells that are responsible for resistance to conventional chemotherapy (Im et al., 2016). Via the stabilization of the transcription factor STAT3 (*signal transducer and activator of transcription 3*) overexpressed BAG3 may confer stem-cell like characteristic on glioblastoma cells. Moreover, the BAG3 protein was found to be specifically overexpressed in endometrioid endometrial adenocarcinomas, suggesting a function of BAG3 in the maintenance of cell survival in uterine cancer (Esposito et al., 2017). BAG3 is also highly expressed in many hepatocellular carcinomas and promotes invasiveness and angiogenesis in these cancer cells (Xiao et al., 2014). In breast cancer cells and in tissue from breast cancer patients, stress resistance and survival after oxidative stress were demonstrated to be linked to an ERα-induced non-canonical autophagy pathway which is, at least in part, mediated by BAG3 (Felzen et al., 2015). In contrast to normal lung tissue, it was found that BAG3 is also overexpressed in lung carcinomas, rendering their resistance to chemotherapy (Chiappetta et al., 2014). The down-regulation of BAG3 in human small-cell lung cancer cell lines was observed to lead to an increased cell death; depletion of BAG3 in an *in vivo* mouse xenograft model significantly reduced tumor growth and induced apoptosis. In breast cancer cells it could be demonstrated that upregulated BAG3 contributes to therapy-induced senescence, thereby preventing apoptosis and mediating chemotherapy resistance (Pasillas et al., 2015). To that end, BAG3 has been shown to interact with MVP (*Major Vault Protein*) and to allow its nuclear accumulation that in turn results in the enhanced activation of the pro-survival ERK1/2 (*extracellular signal-regulated kinase1/2*) signaling pathway. The resistance of rhabdomyosarcoma cells to proteotoxic stress upon concomitant inhibition of PQC systems was reported to depend on the induction of BAG3 by NIK [*nuclear factor-kappa B (NF-κB)-inducing kinase*] (Rapino et al., 2015). In urothelial cancer cells, BAG3 was identified to play a pivotal role in the response to BH3 (BCL-2 homology 3)-mimetics (Mani et al., 2016). BH3-mimetics are peptides or small molecules that mimic the action of BH3-only proteins, therefore representing promising anti-cancer agents (Billard, 2013). As their name implies, “BH3-only proteins” solely contain one BH (BCL-2 homology) domain, namely the BH3 domain; they interact with pro-survival, anti-apoptotic BCL-2 family members like BCL-2 or MCL-1, thereby inhibiting their activity and triggering apoptosis. The depletion of BAG3 resulted in a higher sensitivity of urothelial cancer cells to apoptotic cell death triggered by BH3-mimetics, while the general inhibition of autophagy had no significant effect on cell survival (Mani et al., 2016). Furthermore, the BAG3 expression level was found to be increased in human chronic lymphocytic leukemia (CLL) cells (Zhu et al., 2014). By knocking down BAG3, it could be demonstrated that BAG3 inhibits cell apoptosis in primary CLL cells and promotes their cell migration. Additionally, an evaluated BAG3 expression has been observed to correlate with a poor overall survival of CLL patients, thereby suggesting BAG3 as a marker protein of poor prognostic in particular subgroups of CLL patients. The clinical behavior of a subgroup of stage III melanoma patients has been revealed to be influenced by BAG3 expression (Guerriero et al., 2014). Thus, the analysis of BAG3 expression in melanoma metastatic lymph nodes biopsies was proposed to potentially contribute to cancer prognosis.

In summary, the above listed studies clearly demonstrate that the anti-apoptotic BAG3 protein represents a potential promising target for anti-cancer therapies. In addition, it seems to function as a suitable biomarker for cancer. BAG3 has been reported to act in concert with the HSP70 chaperone in controlling cancer cell signaling; thereby, the HSP70-BAG3 complex was found to modulate the activity of various proteins, such as the transcription factors NF-κB, FoxM1 and Hif1α, the translation regulator HuR and the cell-cycle regulators p21 and survivin. Thus, it is not very surprising that the interaction of HSP70 with BAG3 is considered as a potential therapeutic target in anti-cancer therapy. To that end, small molecules, which disrupt the HSP70-BAG3 interaction, were identified; these compounds revealed anti-proliferative activity in various cancer cells and were able to inhibit tumor growth *in vivo* (Colvin et al., 2014; Li et al., 2015; Antonietti et al., 2017). Notably, the natural compound cantharidin and the dietary flavonoid fisetin were discovered to exhibit anti-cancer activities by indirect suppression of HSP70 and BAG3 expression (Kim J.A. et al., 2013; Kim et al., 2015).

## BAG3-Mediated Selective Macroautophagy

### BAG1-BAG3 Switch in Protein Quality Control

**FIGURE 4:**
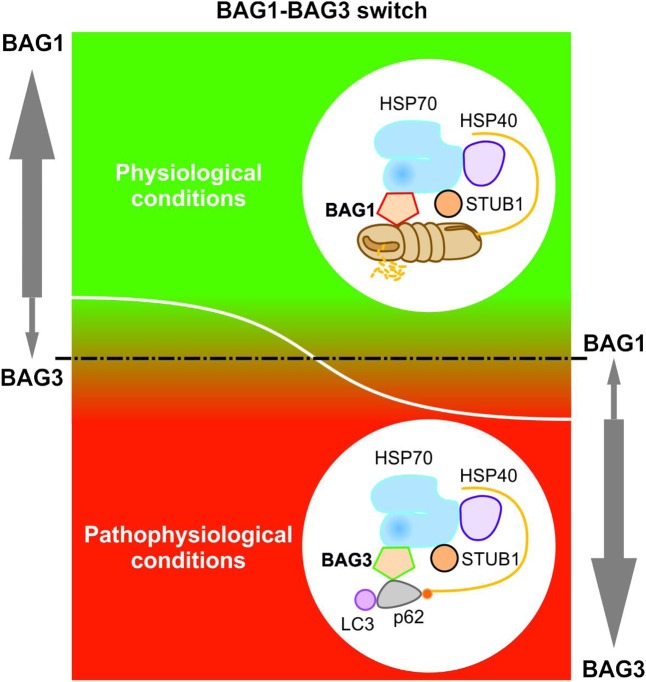
BAG1-BAG3 switch in expression and function. The BAG1 and BAG3 expression levels are reciprocally regulated during cellular aging and under acute stress. Under physiological conditions, a high BAG1 expression, but a low BAG3 expression could be detected; while under pathophysiological conditions, the BAG3 level is elevated and the BAG1 level is decreased. The BAG1 to BAG3 expression switch is accompanied by a functional switch from HSP70-BAG1-mediated proteasomal degradation to HSP70-BAG3-mediated selective macroautophagy.

Elucidating the specific roles of the HSC70/HSP70 co-chaperones BAG1 and BAG3 in cellular protein quality control, we uncovered by using the IMR90 cell model of aging that the BAG1 and BAG3 expression levels are reciprocally regulated during cellular aging and under acute stress; thus, in comparison to young fibroblasts we could detect an increased BAG3 expression, however, a decreased BAG1 expression in aged fibroblasts (Gamerdinger et al., 2009). In addition to an increased BAG3 expression, aged or stressed fibroblasts revealed an elevated autophagic flux and upregulated levels of the polyUb- and LC3-binding protein p62 and of autophagosomal markers like the early autophagosome marker WIPI (Gamerdinger et al., 2009). It has been demonstrated that upon proteasomal impairment BAG3 induces the sequestration of proteasomal clients in cytoplasmic punctae which co-localize with the autophagosomal marker proteins LC3 and WIPI (Gamerdinger et al., 2009; Minoia et al., 2014). Interestingly, relatively higher expression levels of BAG3 and of autophagosomal markers were also found in neurons of the aged rodent brain (Gamerdinger et al., 2009). The BAG1 to BAG3 expression switch is therefore accompanied by a functional switch (**Figure 4**). While BAG1 in a ternary complex together with HSP70 and STUB1 predominantly mediates the degradation of poly-ubiquitinated proteins by the proteasome under physiological conditions (Demand et al., 2001; Alberti et al., 2003), BAG3 triggers the turnover of poly-ubiquitinated proteins by the autophagic-lysosomal system under pathophysiological conditions (Carra et al., 2008a; Gamerdinger et al., 2009, 2011a; Minoia et al., 2014). This BAG3-driven autophagic pathway is referred to as *BAG3-mediated selective macroautophagy pathway* (Gamerdinger et al., 2009, 2011a,b; Behl, 2011, 2016). The switch from a high BAG1-dependent proteasomal activity to a more intensive use of a BAG3-stimulated macroautophagy system for protein degradation represents an adaptive response of the cellular chaperone system to environmental changes. By recruiting a macroautophagy pathway, BAG3 is able to adapt the protein quality control system under pro-oxidant and aggregation-prone conditions. Thus, the BAG3-mediated selective macroautophagy pathway functions as a pivotal safeguarding and emergency system of the cellular protein quality control to maintain cellular protein homeostasis.

The BAG3-mediated selective macroautophagy process seems to be a universal and evolutionary highly conserved cellular mechanism to overcome proteasomal impairment or overload by upregulating BAG3 (Gamerdinger et al., 2009, 2011b; Minoia et al., 2014). In *Drosophila melanogaster* the muscle integrity and muscular protein homeostasis is ensured by an analogous macroautophagy process (called CASA) that is mediated by the functional BAG3 ortholog starvin (see Impact of BAG3 on Myopathies) (Arndt et al., 2010; Ulbricht et al., 2013b).

### The BAG3-Mediated Selective Macroautophagy Pathway in Detail

**FIGURE 5:**
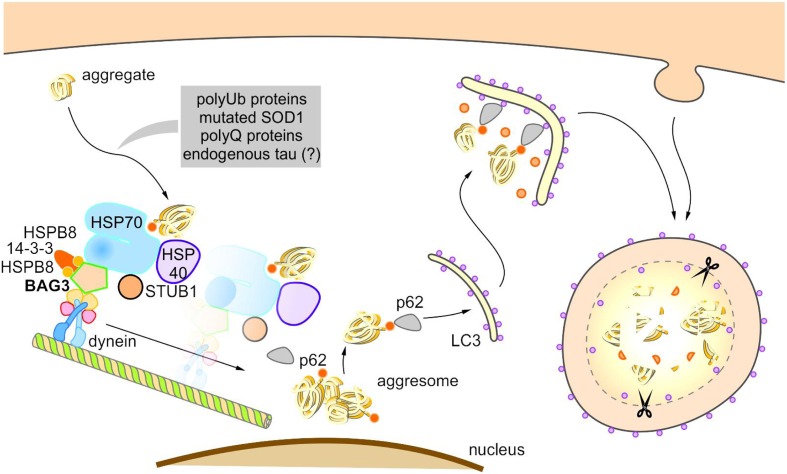
Model of BAG3-mediated aggresome-targeting and selective macroautophagy. During aging and under acute cellular stress, misfolded proteins and/or aggregates bind to a multi-chaperone complex consisting of HSPB8, HSP70, HSP40, BAG3, 14-3-3γ, and STUB1. Subsequently, BAG3 mediates in concert with the 14-3-3γ protein the association of this complex with the dynein complex and thereby initiates the microtubule-based retrograde transport of the degradation-prone substrates to the aggresome. BAG3 induces the selective macroautophagy of the substrates by complexing with the macroautophagy receptor protein p62 that interacts simultaneously with both the substrate as well as the autophagosome membrane-associated protein LC3. The substrates are finally degraded by the autophagic-lysosomal system.

Under pathophysiological conditions, the co-chaperone BAG3 induces selective macroautophagy and thereby in association with the molecular HSP70 chaperone and the autophagy receptor p62 specifically targets aggregation-prone proteins to autophagic degradation (**Figure 5**) (Carra et al., 2008a,b; Gamerdinger et al., 2009, 2011b). Intriguingly, it was recently reported that in glioblastoma cells the activation of selective macroautophagy by overexpressed BAG3 is dependent on its N-terminally located WW domain (Merabova et al., 2015). Furthermore, Rodriguez et al. (2016) showed that BAG3 controls macroautophagy by regulating the basal amount of total autophagosomal marker protein LC3B through a translational mechanism.

The BAG3-stimulated lysosomal degradation of proteins firstly requires the sequestration of aberrant proteins in inclusion bodies by their retrograde transport along microtubules to the microtubule organization center (MTOC) (Gamerdinger et al., 2011b). BAG3 triggers this microtubule-dependent substrate sequestration and thereby facilitates the formation of a perinuclearly located compartment, the so-called aggresome (Johnston et al., 1998; Kopito, 2000; Gamerdinger et al., 2011b; Zhang and Qian, 2011). Aggresomes display a high autophagic activity and are surrounded by a protein cage consisting of the intermediate filament protein vimentin (which therefore serves as an aggresome marker). BAG3-mediated aggresome-targeting is depending on the interaction of BAG3 with the motor protein dynein (Garcia-Mata et al., 1999; Gamerdinger et al., 2011b). We revealed that the PxxP region of BAG3, which is located upstream of the conserved BAG domain, is essential for its binding to dynein. Recently, it has been reported that the 14-3-3γ protein is implicated in the BAG3-dynein interaction by its dimeric binding to the dynein-intermediate chain (DIC) and to BAG3 (to the phosphoserine-containing 14-3-3 binding motifs RSQS^136^ and RSQS^173^); thereby, 14-3-3γ is suggested to act as a molecular adaptor in the BAG3-triggered aggresome-targeting process (Xu et al., 2013). Via its conserved BAG domain and its two conserved IPV motifs, BAG3 can bind to the molecular chaperones HSP70 and HSPB8 (also HSPB6), thereby forming a multi-chaperone complex which additionally contains the ubiquitin ligase STUB1 as well as the HSP40 DNAJB6 (HSPB8-HSP70-BAG3 complex) (Carra et al., 2008b; Gamerdinger et al., 2011b; Sarparanta et al., 2012; Minoia et al., 2014). During BAG3-stimulated aggresome-targeting, BAG3 couples this multi-chaperone complex loaded with the substrate to the cytoplasmic dynein motor complex and in this way induces the release of the chaperone-bound substrate (Gamerdinger et al., 2011b; Minoia et al., 2014).

Based on the so far published studies (Carra et al., 2008b; Gamerdinger et al., 2009, 2011b; Xu et al., 2013; Minoia et al., 2014), the following putative model of BAG3-mediated aggresome-targeting and BAG3-induced selective macroautophagy can be drawn up (**Figure 5**): During aging and under acute cellular stress, aggregation-prone proteins bind to the HSPB8-HSP70-BAG3 chaperone complex. Subsequently, BAG3 in concert with the 14-3-3γ protein mediates the association of this complex with the dynein complex and thereby initiates the microtubule-based retrograde transport of the degradation-prone substrates to the aggresome. BAG3 then induces selective macroautophagy by cooperating with the macroautophagy receptor protein p62 that is able to interact simultaneously with both the substrate and the autophagosome membrane-associated protein LC3. The substrates are finally degraded by the autophagic-lysosomal system. The co-chaperone BAG3 itself is not subject to autophagic degradation during BAG3-mediated selective macroautophagy (Gamerdinger et al., 2009). Interestingly, a substrate ubiquitination (by STUB1 or maybe other E3-ligases) is not necessarily required for the removal of aberrant proteins by the described BAG3-mediated selective macroautophagy pathway; non-ubiquitinated proteins [e.g., the ALS-linked mutant superoxide dismutase 1 (SOD1^G85R^)] have been found to be also targeted for autophagic degradation by this particular macroautophagy pathway (Gamerdinger et al., 2011b).

## BAG3-Pathway in Age-Related Neurodegenerative Disorders

The accumulation and aggregation of intracellular proteins in insoluble inclusion bodies (e.g., aggresomes) in neurons as well as the resulting disturbed neuronal protein homeostasis are key molecular hallmarks of various age-related neurodegenerative disorders, such as Alzheimer’s disease, Parkinson’s disease, Huntington’s disease or amyotrophic lateral sclerosis (Nixon, 2013; Hipp et al., 2014; Ciechanover and Kwon, 2015). The BAG3-mediated selective macroautophagy pathway has been shown to exert a pivotal protective role in cellular protein quality control by degrading upcoming harmful proteins with high aggregation potential (Carra et al., 2008b; Gamerdinger et al., 2009, 2011b; Xu et al., 2013; Minoia et al., 2014). Importantly, the BAG3-pathway is also able to remove disease-associated aggregation-prone proteins, such as mutated SOD1 or polyQ43-huntingtin, and is thereby linked to neuroprotection in age-related neurodegenerative disorders, like ALS, HD, or AD.

### Amyotrophic Lateral Sclerosis (ALS)

Amyothrophic lateral sclerosis is a motoneuron disease that is associated with pathological aggregates of misfolded proteins like mutant forms of SOD1 (*Cu/Zn superoxide dismutase 1*) or TDP-43 (*TAR DNA-binding protein 43*) in affected motoneurons, especially in the motoneurons of the spinal cord (Johnston et al., 2000; Robberecht and Philips, 2013). ALS-linked mutant forms of SOD1, such as SOD1^G85R^ or SOD1^G93A^, have been shown to be selectively degraded by BAG3-mediated selective macroautophagy (Crippa et al., 2010b, 2013; Gamerdinger et al., 2011b). We used mutant SOD1^G85R^ as a model protein to study the mechanism of BAG3-mediated aggresome-targeting and selective macroautophagy (Gamerdinger et al., 2011b). We found that the co-chaperone BAG3 in concert with HSP70 mediates the binding of SOD1^G85R^ to the cytoplasmic dynein complex and thereby specifically directs SOD1^G85R^ to the aggresome. Notably, SOD1^G85R^ coupled to the dynein complex was not ubiquitinated. Aggresomal SOD1^G85R^ was shown to be co-labeled with autophagosomal and lysosomal markers, indicating the autophagic degradation of SOD1^G85R^ (Gamerdinger et al., 2011b). In ALS mice transgenic for SOD1^G85R^ (at disease end stage) an increased BAG3 expression and aggregates positively labeled for both SOD1^G85R^ and BAG3 could be detected in motoneurons of the spinal cord (Gamerdinger et al., 2011b). Crippa et al. (2010a, 2010b, 2013) intensively investigated the impact of the molecular chaperone HSPB8 in cooperation with BAG3 and HSP70 on the clearance of the ALS-linked mutant SOD1^G93A^. In spinal cord motoneurons of transgenic SOD1^G93A^ mice (at the symptomatic stage of disease) they demonstrated an upregulation of BAG3, HSPB8 and HSP70 expression and an enhanced removal of SOD1^G93A^ aggregates by the autophagosome-lysosome system (Crippa et al., 2010b, 2013). The aggresomal sorting as well as the activation of the autophagic clearance of SOD1^G93A^ is thereby mediated by the HSPB8-HSP70-BAG3 chaperone complex (Crippa et al., 2013).

### Huntington’s Disease (HD)

At the molecular level Huntington’s disease is caused by a CAG repeat expansion within the huntingtin gene encoding for an elongated poly-glutamine (polyQ) tract in the huntingtin protein. PolyQ-huntingtin proteins containing a stretch with more than 35 glutamines are highly aggregation-prone and form cytoplasmic aggregates as well as nuclear inclusions in the brain cells which are characteristic for the pathogenesis of HD. In addition to polyQ-huntingtin proteins and amyloid fibers, polyQ inclusions consist of sequestered other cellular proteins, like transcription factors, and therefore exhibit a high gain-of-function toxicity (Labbadia and Morimoto, 2013). Aggregates of the pathogenic mutant form polyQ43-huntingtin (huntingtin protein with 43 glutamines) have been demonstrated to be selectively removed by the BAG3-mediated selective macroautophagy pathway; polyQ43-huntingtin was initially used as model substrate in studies investigating the detailed function of HSPB8 and other sHSP family members in selective macroautophagy (Carra et al., 2005, 2008a,b; Fuchs et al., 2009). HSPB8 revealed chaperone activity toward polyQ43-huntingtin, keeping it in a non-aggregated state competent for degradation (Carra et al., 2005). The co-chaperone BAG3 is crucial for HSPB8 activity in preventing polyQ43-huntingtin aggregates (Carra et al., 2008a). It has been shown that in cooperation with HSPB8 (or also with HSPB6) BAG3 promotes and facilitates the clearance of aggregation-prone polyQ43-huntingtin (Carra et al., 2008a; Fuchs et al., 2009). By knocking down the endogenous HSPB8-BAG3 complex (but not HSPB8 only) a decreased autophagic flux combined with an increased polyQ43-huntingtin aggregation could be detected (Carra et al., 2008a). Notably, neither the deletion of the BAG domain of BAG3 nor the deletion of the WW domain of BAG3 had a negative effect on polyQ43-huntingtin degradation; upon overexpression both mutants decreased the polyQ43-huntingtin aggregation like the full-length BAG3, indicating that BAG3 binding to HSP70 is not essential for polyQ43-huntingtin clearance (Carra et al., 2008a,b). In contrast, the PxxP region of BAG3, the binding site of dynein within BAG3, has been demonstrated to be required for inducing the autophagic degradation of polyQ43-huntingtin.

### Alzheimer’s Disease (AD)

Alzheimer’s disease is neuropathologically characterized by extracellular β-amyloid (Aβ)-containing plaques, intracellular neurofibrillary tangles (NFTs), reduced synaptic density and neuronal loss in affected brain areas (Morawe et al., 2012). The microtubule-associated protein tau was identified to form the filamentous core of the NFTs and to be highly phosphorylated in NFTs (Gotz et al., 2013). Recently, Lei et al. (2015) discovered that in neurons BAG3 may be implicated in the regulation of tau levels. They showed that under stress conditions, such as proteasome inhibition, increased BAG3 expression promotes tau degradation in neurons; vice versa knockdown of BAG3 prevented the decrease in tau levels under these conditions. By exclusively overexpressing BAG3, they were able to drastically reduce endogenous tau and phospho-tau levels in rat primary neurons. The data of Lei et al. (2015) indicate that under stress conditions BAG3 facilitates the removal of soluble tau by its enhanced targeting to selective macroautophagy. In AD the BAG3 expression may not suffice to degrade pathologic forms of the tau protein. Therefore, interventions enhancing the BAG3 expression and thereby the clearance of tau in neurons may be of therapeutic benefit in AD. Interestingly, the cleavage product of the AD-associated amyloid β precursor protein sAβPPα was recently observed to negatively modulate BAG3-mediated aggresome formation; it was able to inhibit the stress-induced upregulation of BAG3 and to enhance proteasomal activity in the cell (Renziehausen et al., 2015; Kundu et al., 2016).

In summary, these findings clearly illustrate that BAG3 may be a key player in neuroprotection by promoting the clearance of aggregated proteins associated with age-related neurodegenerative disorders. Under stress conditions (meaning increased BAG3 levels), disease-related aggregation-prone proteins are specifically directed to the aggresome by the HSPB8-HSP70-BAG3 complex and subsequently degraded by BAG3-induced selective macroautophagy. Notably, Seidel et al. (2012) observed a cell-type specific upregulation of BAG3 and HSPB8 in astrocytes that are located within human brain areas affected by neurodegeneration; they could not detect elevated levels of BAG3 and HSPB8 in the affected neurons. The increased levels of BAG3 and HSPB8 in astrocytes were claimed to possibly facilitate the degradation of debris (from dead neurons) and extracellular aggregated proteins. Intriguingly, 14-3-3 proteins have been found to be part of inclusion bodies in a number of neurodegenerative diseases, proposing a role of 14-3-3 in promoting the formation of aggresomes during neurodegeneration (Zhao et al., 2011; Xu et al., 2013; Jia et al., 2014; Aghazadeh and Papadopoulos, 2016). Based on these results, the enhanced specific clearance of disease-related aggregate-prone proteins seems to be a therapeutic strategy and is indeed under discussion to be a potential therapeutic approach in neurodegenerative disorders (Seidel et al., 2012; Hochfeld et al., 2013; Ciechanover and Kwon, 2015; Eisele et al., 2015; Lei et al., 2015).

## Conclusion and Future Prospects: BAG3 as a Novel Therapeutic Target?

The cellular activity of the HSP70 co-chaperone BAG3 is mainly characterized by its anti-apoptotic function as well as by its role in selective macroautophagy. On the one hand, BAG3 seems to stimulate the development, invasion and metastasis of cancers by sustaining cell survival and resistance to chemotherapy; on the other hand, under proteasomal impairment or overload (e.g., under acute stress, during aging, or disorders) it mediates the disposal of degradation-prone proteins and/or aggregates by triggering selective macroautophagy. To that end, an elevated BAG3 expression might be of advantage for the removal of disease-related protein aggregates; in contrast, BAG3 overexpression also triggers its anti-apoptotic activity and is therefore stimulating proliferation of neoplastic cells. The control and regulation of *bag3* gene expression are still not understood in detail and are currently subject of intense research. Many recent studies discuss BAG3 as a potential promising target not only for anti-cancer therapy, but also for the therapy of diseases like neurodegenerative disorders or myopathies. Thereby, BAG3 expression not only has to be activated selectively, but also regulated cell type-, tissue-, age-, and disease-specifically. In addition to the control of BAG3 expression on transcriptional and/or translational level, the interference with BAG3-protein interactions is considered as potential therapeutic approach. The application of small molecules/compounds might represent a suitable tool not only to selectively induce or suppress BAG3 expression, but also to directly and specifically disrupt or enhance particular BAG3-protein interactions, thereby directing certain processes; however, the identification of such compounds is a future big challenge.

## Author Contributions

ES designed the review and the figures and wrote the paper. CB designed and wrote the paper.

## Conflict of Interest Statement

The authors declare that the research was conducted in the absence of any commercial or financial relationships that could be construed as a potential conflict of interest.
